# A Text-Book of Practical Medicine

**Published:** 1884-12

**Authors:** 


**A Text-Book of Practical Medicine.** Designed for the use of Students and Practitioners of Medicine. By Alfred L. Loomis, M. D., LL. D., Professor of Pathology and Practical Medicine, Medical Department University of the City of New York. With 211 illustrations. New York: William Wood & Co.

The national reputation which Dr. Loomis enjoys would alone ensure the success of this book, but even a somewhat hasty examination of it assures us that his work does not need the prestige of a great name. Independent of the author's reputation, the book would command a position as one of the very best of its kind. The skill and learning of a great clinician are manifest in every part of the book. The author states that it is practically a revision and elaboration of his lectures, given during the last eighteen years, and it bears the marks of that origin, not only in its direct and practical bearings and the business and vigor of its style, but also in drawing the outlines of general principles in a dogmatic way, avoiding all discussion of unsettled questions. The abundance of illustrations representing the morbid changes and objective symptoms of disease is a commendable peculiarity of the book. The publishers have presented it in most attractive form, and we believe it is destined to be the most popular work in practical medicine yet published.

